# US public and private payer coverage for tobacco cessation treatments and barriers to accessing treatments

**DOI:** 10.18332/tpc/149483

**Published:** 2022-05-17

**Authors:** Radha Hussain, Jennifer H. LeLaurin, Ramzi G. Salloum

**Affiliations:** 1 College of Public Health and Health Professions, University of Florida, Gainesville, United States; 2 Department of Health Outcomes and Biomedical Informatics, College of Medicine, University of Florida, Gainesville, United States

**Keywords:** tobacco use cessation, insurance, health policy

**Dear Editor,**

Smoking is a primary cause of preventable morbidity and mortality in the US^1^. Use of evidence-based smoking cessation medications increases the likelihood of quitting; however, less than one-third of US smokers making a quit attempt use these medications^2^. The U.S. Public Health Service (USPHS) issued recommendations to treat tobacco dependence as a chronic disease that requires repeated interventions and multiple quit attempts with existing effective treatments^3^. Further, the USPHS calls for insurance coverage of cessation medication free of copays, coverage limits, limits on quit attempts, and prior authorizations.

Access to cessation medications increased with the enactment of the Affordable Care Act (ACA) in 2014, which required most insurance plans to cover cessation medications^4,5^. Unfortunately, smokers still face obstacles to obtaining coverage of these medications due to insurance plan limitations. Each state’s Medicaid program publishes criteria for coverage of tobacco cessation treatments. This information is also available for large private insurance plans, such as Anthem Blue Cross Blue Shield (BCBS) which is common to all US states.

To identify barriers to accessing cessation medications, we reviewed coverage restrictions of Medicaid and BCBS payers, including prior authorization requirements, annual limits on duration, and limits on quit attempts. We found that all plans required a prescription for any of the covered smoking cessation medications, even for over-the-counter drugs. When comparing cessation coverage between these two plans across all states, there were more restrictions found with Medicaid than commercial BCBS (Table 1). Notably, only 9 (17.6%) of state Medicaid plans provided unrestricted coverage compared to 20 (39.2%) BCBS plans.

**Table 1:** Comparison of cessation medication coverage for Medicaid and BCBS for US states and Washington, D.C.

*Coverage criteria*	*Medicaid plans n (%)*	*BCBS plans n (%)*
**Prior authorizations for non-preferred medications**	23 (45.1)	13 (25.5)
Chantix	2 (3.9)	7 (13.7)
Nicotrol nasal spray	21 (41.2)	11 (21.6)
Nicotrol inhaler	21 (41.2)	11 (21.6)
**Prior authorizations for treatment extension**	3 (5.9)	1 (2.0)
**Counseling required**	8 (15.7)	0 (0.0)
**Duration of therapy limits**	36 (70.6)	18 (35.3)
**Prior authorization required for treatment >90 days**	8 (15.7)	1 (2.0)
**Prior authorization required for treatment >180 days**	25 (49.0)	17 (33.3)
**Stepped-care therapy**	3 (5.9)	0 (0.0)
**Annual limits on quit attempts**		
0	15 (29.4)	30 (58.8)
1	5 (9.8)	1 (2.0)
2	30 (58.8)	20 (39.2)
3	1 (2.0)	0 (0.0)
Unlimited coverage^a^	9 (17.6)	20 (39.2)

a No prior authorization requirements, limits on duration, or limits on quit attempts.

The 2020 U.S. Surgeon General’s report recommended that health plans increase the availability and promote the use of evidence-based cessation medications, leading to higher rates of successful quitting^2^. Despite this recommendation, our findings show health plans have maintained restrictions on cessation treatments a nearly decade after passage of the ACA. The average smoker will make 8–11 attempts to quit before he/she is successful^6^, making the annual quit attempt restrictions found in 36 (70.1%) Medicaid and 21 (41.2%) BCBS state plans unreasonable. Prior authorization requirements for specific medications found in 23 (45.1%) Medicaid and 13 (25.5%) BCBS plans create further obstacles, as many smokers and their physicians may be unaware of how to navigate through the prior authorization process. Such barriers can lead to delays in treatment for individuals who are ready to quit. Limits on treatment duration present in 36 (70.6%) Medicaid and 18 (35.3%) BCBS plans may lead to relapse in individuals who need a longer period of support to achieve abstinence.

Despite extensive evidence on the benefits of smoking cessation treatments and efforts to increase access to cessation medications, major barriers to coverage persist among public and private payers in the US. More policy work is needed to ensure that health plans are providing comprehensive, unrestricted coverage of smoking cessation medications.

## Data Availability

Data sharing is not applicable to this article as no new data were created.
